# Use of Short-Term CIDR-Based Protocols for Oestrus Synchronisation in Goats at Tropical and Subtropical Latitudes

**DOI:** 10.3390/ani14111560

**Published:** 2024-05-24

**Authors:** Angella Nakafeero, Antonio Gonzalez-Bulnes, Paula Martinez-Ros

**Affiliations:** Departamento de Produccion y Sanidad Animal, Facultad de Veterinaria, Universidad Cardenal Herrera-CEU, CEU Universities, C/Tirant lo Blanc, 7, Alfara del Patriarca, 46115 Valencia, Spain; antonio.gonzalezbulnes@uchceu.es

**Keywords:** oestrus synchronisation, CIDR, goats, tropical, subtropical

## Abstract

**Simple Summary:**

There is ongoing research regarding the application and efficiency of short-term oestrus synchronisation (ES) treatments using controlled internal drug release (CIDR) in goats raised under subtropical environments, but information regarding the extent of application of these protocols in the tropical region is limited. Although short-term CIDR-based protocols have been more widely studied and applied in the subtropical region, they are still challenged by ethical, technical, and economic issues. Tropical and subtropical regions have moderately seasonal to aseasonal goat breeds, and so, efficient protocols involving a reduced hormonal supply can be developed for the management of reproduction. Therefore, this review provides a scope of the application of short-term CIDR-based protocols and co-treatments and suggests areas of improvement.

**Abstract:**

This review aims to provide an insight into the application and efficiency of CIDR-based protocols for ES in goats raised under tropical and subtropical environments. In temperate regions, short-term CIDR treatments are replacing long-term treatments and sponges used in earlier decades. In addition, the use of co-treatments for the induction of ovulation is gradually changing from hormonal to non-hormonal methods, given the drive towards clean, green, and ethical techniques for reproductive management. Whereas the subtropical region registers ongoing research in the development of new ES protocols, there are few reports from the tropics, particularly Africa, one of the regions with the highest population of goats. Therefore, this calls for research to develop the most appropriate protocols for these regions, since the protocols currently used are largely hormonal based, as they were developed for goats at higher latitudes. Management and environmental factors determine the breeding pattern of goats at tropical latitudes rather than photoperiods, and they are the main causes of reproductive seasonality. The use of ES methods, particularly short-term CIDR-based protocols, along with artificial insemination, may have a significant impact on the productivity of goats in these regions when these factors are controlled.

## 1. Introduction

The world’s goat population is currently estimated at 1.6 billion [1], and it is mainly distributed in Asia, Africa, and South America [2]. These regions have a wide diversity of goat breeds, with traits for production of milk, meat, skin, and hair [3]. Goat breeds, particularly those originating at subtropical latitudes (25–35°) and higher, exhibit a seasonal pattern of reproduction [4]. On the other hand, breeds of tropical origin are generally considered as continuous breeders [5]. In recent years, extensive importation of genotypes from temperate and Mediterranean latitudes has introduced breeds with seasonal breeding patterns; therefore, goat breeds under tropical latitudes also exhibit reproductive seasonality [6]. Thus, to ensure a continuous pattern of food production, the use of ES is essential, both under subtropical and tropical latitudes, as a reproductive management tool for synchronising breeding and kidding [7] and for the control of reproductive seasonality [8]. ES is also a useful tool for propagating improved genetics when used alongside artificial insemination [9]. Research aims to reduce residues when different types of intravaginal devices are used [10]. Accordingly, use of devices containing lower doses of hormones, and the use of natural as opposed to synthetic hormones, is promoted, thus favouring natural methods for ES [11]. Secondly, aspects related to animal welfare, health, and reproductive efficiency after artificial insemination are critically considered [12]. Therefore, progestogen-impregnated sponges are being replaced by natural-progesterone in CIDR devices, whereas short-term protocols (5–9 days) are substituting long-term protocols (12–21 days).

Artificial insemination makes the use of equine chorionic gonadotrophin (eCG) necessary for a narrow synchronisation of oestrus to be achieved. However, the availability and use of eCG is challenged by ethical concerns surrounding its production, food safety issues, and the fact that its repeated use induces anti-eCG antibodies, which reduces fertility after subsequent treatments. For these reasons, natural methods have been proposed as alternatives to eCG for synchronising ovulation after ES [13,14]. There are reports from subtropical regions [15,16,17] indicating an adequate response to these developments, but limited work is available from the African tropical region, which in fact presents less challenges with regards to reproductive seasonality [15]. Hence, this would be an interesting region for the development of efficient ES protocols for FTAI involving a reduced hormonal supply, given the drive to use greener and cleaner ES methods [18]. Therefore, the aim of this review is to present a scope of the application of ES in the tropical and subtropical regions, with special focus on short-term CIDR-based protocols and hormonal and non-hormonal co-treatments.

## 2. Effectiveness of CIDR Compared with Intravaginal Progestogen-Impregnated Sponges

**Figure 1:**
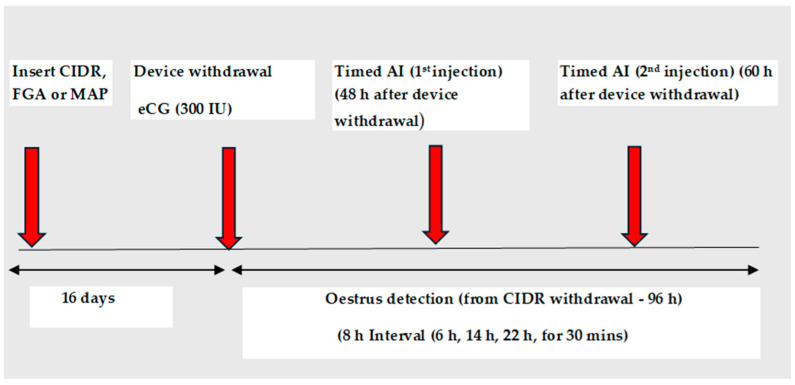
Schematic representation of an ES protocol comparing use of CIDR, MAP, and FGA on South African Boer and indigenous goats. (Based on the study of Motlomelo et al. 2002 [25]).

**Figure 2:**
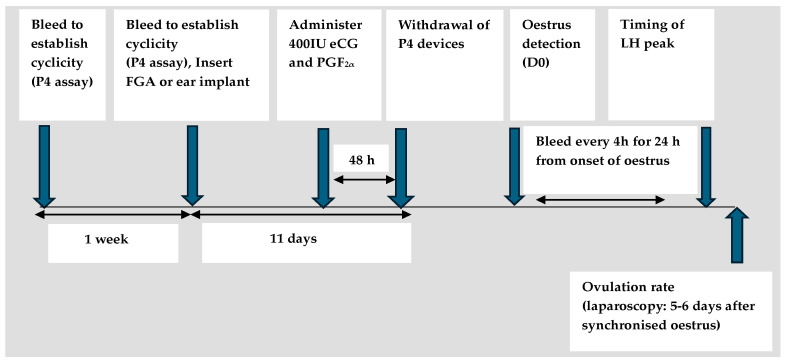
Schematic representation of an ES protocol to evaluate efficiency of FGA or norgestomet ear implants on occurrence and synchronisation of oestrus, LH peak, and ovulation rate in cyclic goats. (Based on the study of Freitas et al. 1997 [32]).

A comparison of different intravaginal devices under various climatic conditions and/latitudes has revealed that the type of intravaginal device (Figure 1 and Figure 2) does not affect the proportion of females expressing oestrus (oestrus response), but several authors have observed a tighter synchrony of oestrus treated with CIDR compared with those treated with Medroxyprogesterone acetate (MAP) and fluorogestone acetate (FGA) sponges [19,20,21]. A high oestrus response (over 90%) has been reported in goats treated at different latitudes during the breeding season, with or without eCG use [21,22,23], and in the non-breeding season, when eCG is administered [24]. These data indicate that both sponges and CIDR are equally effective when used to synchronise oestrus in goats under different climatic conditions. Contrary to oestrus response, there are conflicting reports with regard to the timing of the onset of oestrus, when CIDR or sponges are used. Some authors who compared the use of these devices reported a shorter interval from device withdrawal to oestrus (interval to oestrus) in goats treated with sponges compared with CIDR, whereas [25] reported a shorter interval to oestrus in goats treated with CIDR than sponges (27.2 ± 0.4 vs. 30.9 ± 0.4, *p* < 0.05), which is similar to what is reported in sheep [26,27].

Results in sheep [26] also observed a shorter (*p* < 0.05) interval from device withdrawal to the preovulatory luteinizing hormone (LH) peak, the interval from oestrus to the LH peak, and the duration of oestrus in goats treated with CIDR than in the group treated with sponges. The disagreement in reports with regards to this parameter could be related to differences in the mechanisms of the release/absorption of progesterone/progestogen from CIDR [22] or sponges [25], respectively, which may lead to differences in the control of ovarian follicular dynamics among treated females. Differences in the reproductive responses observed with the use of CIDR or sponges could also be related to the amount of progestogen absorbed by females treated with progestogen sponges, which was reported as being lower than the concentration of hormone contained in the sponges [28,29]. Using 13 cyclic goats, Ref. [28] demonstrated that there are individual differences in the amount of the hormone absorbed from progestogen sponges, as shown by the data on the individual uptake of the hormone by goats. The data indicate variability in the uptake of progestogen, which ranged from 18 to 54 mg. In addition, the study showed that the goat which had the least uptake of progestogen (20.00 ± 0.00 mg), and the highest amount of residual progestogen in the sponge (42.0.0 ± 0.00 mg), was unmated. A significant difference was also observed in the amount of progestogen absorbed in relation to pregnant and non-pregnant goats (26.44 ± 6.06 mg vs. 42.00 ± 10.58 mg, respectively, *p* < 0.01). Similarly, the residual amount of progestogen in the sponges of pregnant and non-pregnant goats was significantly different (35.56 ± 6.06 mg vs. 20.00 ± 10.58 mg, respectively, *p* < 0.01). This gives the impression that variability in reproductive responses observed in females treated with sponges could be attributed to individual differences in the absorption of progestogen.

In view of these conflicting reports concerning the interval to the onset of oestrus, we should focus on the need to determine the correct timing for AI, given the variability associated with the timing of the LH peak when different ES methods are used, which in turn affects the timing of ovulation [22] and fertility when AI is applied [30].

This consideration is supported by the fact that fertility results reported when insemination is performed based on the interval of the onset of oestrus [25,31], or at fixed times after the observation of oestrous signs are variable, both in treated goats and sheep [32,33,34]. For example, Ref. [32] obtained high conception (76.8%) and kidding rates (75%) in anoestrus goats inseminated 24 h after the observation of oestrous signs, whereas [34], who used a similar protocol on anoestrus sheep, reported poor fertility yields of 43.47% (22/50). Poor oestrus response (70–80%) and fertility (45–52%) have also been reported when the insemination was performed 12 h after the onset of oestrus in sheep during the breeding season. Fertility results reported in goats after performing double inseminations at 48 h and 60 h intervals to oestrus [25,31] also demonstrate variability in reproductive responses. Besides fertility traits, there is also a need to consider the effects of the device on animal welfare and health. High incidences of clinical vaginitis have been reported in goats [35,36,37]. An increase (*p* < 0.001) in the number of colony-forming units (CFUs) was detected by [35] in vaginal samples collected from females treated with sponges. The authors observed that only 6.1% (2/33) of vaginal samples collected from goats treated with sponges had colony-forming units (CFUs ≥ 10^5^), with the most prevalent bacterium belonging to the genus *Staphylococcus* sp., whereas at sponge withdrawal, CFUs ≥ 10^5^ were detected in 62.5% (18/29) of the collected samples, with *Escherichia coli* being the most prevalent bacterium. However, few studies demonstrate that there is an effect of vaginitis on fertility, given the observation that fertility rates above 60% are reported after long- and short-term treatments using sponges [21,31,38]. Therefore, vaginitis does not seem to be a major problem with regards to fertility when sponges are used. However, animal welfare and the cost of treating vaginitis would reduce the preference for the use of sponges over CIDR. Other limitations associated with the use of sponges include the risk of losing the inserts during the treatment period [39], which may require constant monitoring of the treated females. Low retention rates of vaginal devices may affect fertility through variability in reproductive responses, whereas a high retention rate increases the proportion of females responding with oestrus signs [40]. The treatment of 288 sheep on 3 farms with the short-term CIDR protocol resulted in a high retention rate of the device (96.5%), with 95.7% of the treated females responding with oestrous signs [40]. A comparison of sponges to CIDR reveals a high retention rate of both devices (100%, Eazi-breed^TM^CIDR^®^G vs. 99%, Chronogest^®^CR [39]), whereas Ref. [26] reported a drop rate of 13.5% for CIDR compared with 6.7% for sponges in Karakul ewes. Although there is a risk of sponges being dropped during treatment, data show that device retention does not seem to be a major challenge to reproductive success. Also, there have been efforts to reduce the impact of residual progestogen in sponges on the environment. High levels of residual progestogen have been detected in previously used sponges from goats (32.46 mg ± 9.84 mg [28]) and ewes (25.00 ± 0.84 mg [29]), whose data confirm that the amount of progestogen absorbed by treated females is lower than the dose contained in the sponges [33]. This finding therefore supports the use of sponges containing lower doses of progestogen and is backed by reports indicating that a reduction in the progestogen dose from 60 to 30 mg has no effect on ovarian follicular dynamics and function [41] or fertility yields [27,31]. Hence, the use of high progestogen doses seems to be unnecessary. Notwithstanding, studies undertaken to evaluate the effectiveness of CIDR, an intravaginal device developed in the 1980s, which contains 0.3 g of natural progesterone and can be re-used, have contributed much to the current shift from the use of intravaginal sponges to CIDR.

## 3. Effectiveness of Previously Used CIDR Devices

One of the advantages of short-term CIDR treatments in small ruminants is the opportunity to re-use the CIDR device, which offers an economically feasible protocol [13,42]. Therefore, most studies relating to the effectiveness of short-term CIDR treatments have also evaluated the success of re-used CIDRs to induce reproductive responses and fertility in treated females [13,24,43]. These evaluations include new CIDRs compared with CIDR devices previously used once or twice after treatment durations of 5–9 days and the effect of autoclaving or disinfecting previously used CIDRs on fertility parameters. The re-use of CIDR devices reduces the concentration of P4 [24,43], which may expose follicles to subluteal P4 levels during the treatment period [44], thus affecting follicular turn-overs [45]. and fertility [24,43]. The treatment of ewes with either new or re-used CIDRs reveals that although P4 concentrations in both new and used devices are maintained above 1 ng/mL during the treatment period, a peak of the P4 concentration (above 2 ng/mL) is only observed in females treated with new CIDRs [40]. A high peak of the P4 concentration stimulates a high follicular turn-over [46], which results in the ovulation of young follicles and high fertility [45]. Hence, a tendency to ovulate follicles from a new follicular wave was observed by [24], who noted that all goats treated with new CIDRs ovulated from a new follicular wave, compared with 80% of goats treated with re-used CIDRs *(p* = NS). In this study, maximum P4 concentrations detected at 12 h in goats treated for 5 days with a CIDR of first, second, and third use were 19.0 ± 7.3, 10.3 ± 1.9, and 7.7 ± 2.4 nmol/L, respectively, (*p <* 0.05). Accordingly, a significant difference in fertility between goats treated with new and third-use CIDRs (75.3% vs. 62.1%, respectively, *p <* 0.05) was observed.

Strategies such as autoclaving and disinfection, commonly used to sterilise previously used CIDR devices, can also affect the P4 concentration. Ref. [13] observed that anoestrus goats treated with a 14-day previously used autoclaved CIDR tended to have a higher *(p =* 0.06) P4 concentration, compared with goats treated with re-used disinfected devices [13]. In spite of these observations, Ungerfeld and col. [45] did not find any effect of autoclaving previously used devices on the fertility of anoestrus ewes. Generally, the effect of re-used CIDRs on fertility has not been reported by many researchers [13,42,45,46,47,48]. Hence, the re-use or autoclaving of preciously used CIDRs does not seem to affect fertility, although cases where the effect on fertility has been observed may reflect an effect of the duration of previous use or the season, since during the anoestrus season, the fertility of breeding males is also compromised. Therefore, the duration of previous use and the season of treatment may determine fertility outcomes in females treated with re-used short-term CIDR devices. Accordingly, ref. [49], who treated anoestrus ewes with a previously used CIDR for 22 days, obtained poor oestrus response and fertility. Based on the literature, it can therefore be inferred that the treatment of cyclic and anoestrus females with short-term CIDRs previously used for a period not longer than 14 days should result in a high oestrus response and acceptable fertility.

## 4. Effectiveness of Short-Term CIDR Protocols with or without eCG

Equine chorionic gonadotrophin is a glycoprotein hormone secreted by the trophoblastic cells of pregnant mares [50]. It has FSH and LH bioactivity and functions in a similar way, even when used in other species [51]. Due to this outstanding capacity, it is commonly used to induce and synchronise oestrus and ovulation [9], and when administered in high doses (450–600 IU), it increases the ovulation rate by reducing the rate of atresia of preovulatory follicles [52]. Acceptable reproductive outcomes have been obtained when goats are treated with short-term CIDR–eCG protocols associated with natural mating or FTAI [9,16]. This is due to the benefits of eCG use, such as a tight synchrony of oestrus [53], an earlier LH peak, and synchronised ovulations [22], which lead to high fertility, particularly when FTAI is applied [24]. Short-term CIDR–eCG protocols effectively induce oestrus and ovulation, as studies in goats show that over 80% oestrus and ovulatory response can be achieved when females are treated in different seasons [23,24]. Considering the drive to develop ES methods, which are “clean, green and ethical” [18], results show that it is possible to achieve 100% oestrus and ovulatory response when eCG doses as low as 100–200 IU are combined with the short-term CIDR protocol or even without eCG. For example, 100% oestrus response and kidding rates have been reported in cyclic goats under tropical latitudes with a low dose of 100 IU [47], whereas doses of 200 IU, administered during the breeding season [23] at tropical latitudes, result in fertility yields comparable to fertility obtained with higher doses applied at higher latitudes. Furthermore, a low dose of eCG (200 IU), combined with progestins (CIDR and MAP) and administered during the non-breeding season, sufficiently induced oestrus behaviour in a high proportion of goats at temperate, Mediterranean, and subtropical latitudes [24,54], respectively, and resulted in adequate fertility (over 60%), although goats at lower latitudes show a better oestrus response (over 85%) and higher fertility.

The effect of not combining short-term CIDR with eCG on oestrus response and fertility has been observed with the 9-day CIDR protocol [49]. In this study, the oestrus response of Saanen goats and their kidding rates after controlled natural mating (hand mating), with and without eCG were 100% vs. 70%, *p =* NS and 100% vs. 69.8%, *p ≤* 0.05, respectively. In sheep, use of the 9-day CIDR protocol combined with the ram effect showed a high oestrus response (97.5%) and conception rate at 45 days after FTAI (81.0%); however, very low lambing rates (42.9%) were obtained. The protocol used in this study did not involve prostaglandin (PGF_2_α) administered at the end of progesterone treatment, which could have been the cause of low lambing rates in the Ramshort treatment group. The administration of PGF_2_α reduces the progesterone concentration by inducing luteolysis [22] and can be administered either at device insertion [43,55] or withdrawal [48]. Thus, low levels of P4 are induced, and an increase in LH secretion ensure that follicles grow under a high LH and a low P4, which may improve the quality and development of the follicle [47,56] or affect oocyte developmental competence [57], fertilisation rates, or embryo development at later stages of the pregnancy [58]. On the other hand, a high P4 concentration at the end of the treatment would delay the onset of oestrus and also cause variability in the occurrence of preovulatory events among treated females [22], which is undesirable for FTAI. On a similar note, an effect on fertility has been reported when ewes were treated with sponges for 6 days, combined with or without eCG (58% vs. 87%, *p ≤* 0.05) but with PGF_2_α excluded [46]. The CIDR 9-day protocol has not been widely researched, but the most commonly used short-term treatment durations (5, 6, and 7 days) have resulted in similar reproductive outcomes both in goats [23,24] and sheep [59].

The administration of eCG has been reported to advance the onset of oestrus by more than 5 h, advance the LH peak and ovulation [22], and to narrow the interval from device withdrawal to ovulation (interval to ovulation) [40]. It has been described that eCG administration at doses of 400 IU and below does not influence the ovulation rate, fertility, [59] or prolificacy [47]. The onset of oestrus in goats occurs between 27 and 31 h, when the short-term CIDR protocol is applied in goats [22,23,43], whereas it appears to occur much later in sheep (34–40 h) [40,60]. On the other hand, the duration of oestrus signs, which may be used as an indicator of the timing of ovulation, lasts for about 32 h and does not differ in goats and sheep, although longer durations have been observed where low doses of eCG (100 IU) were used [47]. A shorter duration of oestrus signifies an early decrease in the oestradiol (E2) concentration, particularly among eCG-treated females, which advances the LH surge [22]. The interval to ovulation occurs at around 60 h [22] but varies based on whether or not eCG is included, the eCG dose used, and the administration of PGF_2_α and the timing of its application, and these factors influence the interval to oestrus, the timing of the LH peak, and, consequently, the interval to ovulation, which influences fertility at FTAI.

## 5. Limitations of eCG Use and Alternatives and Challenges Associated with the Non-Breeding Season

### 5.1. Limitations of eCG Use

Despite its effectiveness, eCG has the following limitations. (a) There are ethical concerns surrounding its production, which may threaten its future availability [12]. (b) The use of oestrogenic compounds in food-producing animals has been restricted in certain regions and, in general, the use of other hormonal products is under scrutiny and strongly confronted by public opinion [61]. (c) The repeated use of eCG leads to an accumulation of residual antibodies from previous treatments [62,63], which results in reduced fertility with subsequent breeding [64]. This is a major drawback, because once fertility is affected, especially in genetic selection programs, progress too is affected; therefore, the decision to begin using eCG on a flock appears to be risky. However, since several studies indicate that short-term CIDR protocols without eCG result in a similar oestrus response and fertility as protocols combined with eCG [21,22,23], further research is necessary to improve short-term CIDR protocols that can effectively control ovarian follicular dynamics and enhance endogenous gonadotropin secretion after P4 withdrawal. In view of these considerations, the need for eCG use can be eliminated in the near future [22]. Alternatively, studies focused on developing effective short-term CIDR protocols, combined with the gonadotrophin-releasing hormone (GnRH), the male effect (ME), or short-term nutritional supplementation for use in both seasons, particularly at FTAI, would reduce the dependency on eCG.

### 5.2. GnRH as an Alternative to eCG

**Figure 3:**
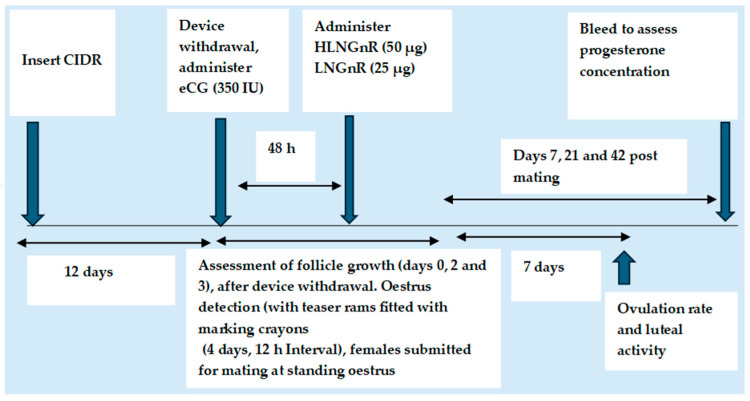
Schematic representation of an ES protocol to compare 2 doses of nano-GnRH with eCG in a long-term CIDR-based protocol applied to anoestrus ewes. (Based on the study of Hashem et al. 2023 [70]).

Research into alternative gonadotrophins such as GnRH to replace eCG use in a short-term CIDR protocol has been undertaken in goats under subtropical latitudes [65], but more work has been conducted in sheep, with the results showing similar reproductive outcomes to eCG [60]. In ewes, the growth of a new follicular wave was observed in 70% of the synchronised females treated with GnRH [66], which was higher (*p* = NS) than the group treated with eCG. Therefore, GnRH can be considered as a possible alternative to eCG, although it has been reported that oestrus expression is highly compromised during the non-breeding season [65,67]. The gonadotrophin-releasing hormone has a low molecular weight and a short lifespan, and these aspects restrict its delivery to the target sites and thus affect its biological activity [68]. In response to this, a recent study in goats synchronised with the ovsynch protocol during the non-breeding season applied GnRH using the nano-delivery system (Figure 3), which induced a tighter synchrony of ovulation and better luteal function [69]. The authors also compared nano-GnRH to eCG in a long-term CIDR-based oestrus synchronisation protocol in sheep and observed an increase in conception, lambing, and fecundity rates (*p* < 0.05), when a high dosage of 50 µg was used [70]. In addition, a tendency of a higher litter size (*p* = 0.081) compared with eCG was obtained. Unpublished data from our research group, applying nano-GnRH combined with a short-term CIDR treatment in Murciano-Granadina goats during the non-breeding season, did not yield good reproductive responses. This therefore calls for more research to improve short-term CIDR protocols combined with GnRH, particularly for out-of-season breeding. Out-of-season breeding is a big challenge. It has been demonstrated that the season in which ES is performed has a significant effect on the timing of the onset of oestrus, the LH surge, and ovulation after withdrawal of the intravaginal device [71]. This is related to the fact that the rate of follicle growth and development is slower during the non-breeding season than in the breeding season, due to the absence of LH stimulation [56,71,72]. It is for the above reason that administration of eCG during the non-breeding season requires higher doses [34,73], above 400–500 IU compared with 200–300 IU, commonly used during breeding season [23,24], which is uneconomical.

### 5.3. Male Effect as an Alternative to eCG

**Figure 4:**
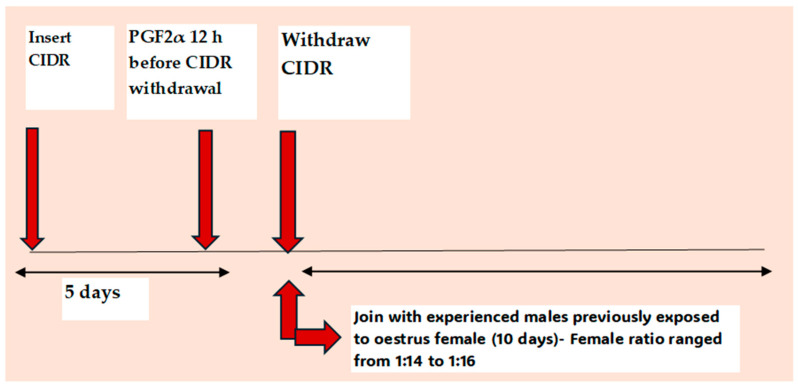
Schematic representation of a short-term CIDR protocol (using a new CIDR), combined with male effect and natural mating, applied to anoestrous dairy goats. (Based on the study of Alvarez et al. 2013 [13]).

The male effect (ME) is another alternative method to eCG use and has been combined with short-term CIDR protocols in goats at tropical, subtropical, and at higher latitudes during the breeding and non-breeding seasons [13,74]. It is more commonly used during the non-breeding season for the induction of ovulation and can be combined with hormonal treatments (Figure 4) or used without the prior hormonal treatment of females [74]. A high oestrus response (97.5%) and adequate fertility (62.5%) are reported in goats where short-term CIDRs combined with ME were used during the non-breeding season [13], whereas a similar protocol produced a lower oestrus response (56.7%) and fertility (50%) in anoestrus ewes [45].

Reproductive responses, particularly during the non-breeding season, can vary due to a number of factors, with the main factor being the type of breed, in relation to its response to photoperiodic changes [4]. Photostimulation can be applied as a natural method of stimulating male sexual behaviour [75], although this does not guarantee high responses among females, particularly when they are not pre-treated with progesterone before exposure [76]. The application of ME to anoestrus goats without pre-P4 treatment has produced low kidding rates (53.3% vs. 73.0%, *p =* NS) and fecundity (66.7% vs. 115.4%, *p <* 0.05) in untreated goats compared with pre-P4 treated goats, respectively [77]. On the contrary, less-seasonal Mediterranean goats exposed to photostimulated bucks during the anoestrus season show higher reproductive responses when exposed to photostimulated bucks without pre-P4 treatment. Ref. [78] found a high proportion of goats ovulating (95%) and achieved an 85% oestrus response. The intervals from male introduction to oestrus and ovulation were 6.9 ± 0.7 days and 10.1 ± 0.7 days, respectively, whereas the obtained fertility (60%) and the number of kids born (1.58 ± 0.15) were comparable to results for hormonal methods. Sexual activity in males can also be stimulated by females in oestrus; therefore, the requirement for a long period of light treatment (about 60 days) can be foregone. For example, the treatment of anoestrus goats with a protocol consisting of a single 25 mg of a P4 injection combined with a low eCG dose (240 IU) during the non-breeding season induced oestrus in anoestrus goats, which were in turn used to stimulate sexual activity in bucks and ovulation in goats treated with a single P4 injection when males and females were joined [79]. As a result, high pregnancy (83%) and kidding (63%) rates were achieved in this study. Photostimulation of both males and females can produce even greater fertility outcomes during non-breeding, as demonstrated in sheep by a high lambing percentage (94.4%), which was obtained within a period of 35 days under a temperate latitude [80]. Hormone-free protocols associated with FTAI have also been researched and have produced adequate fertility, which shows that there has been progress towards the development of greener ES methods. ES protocols based on the use of ME alone have proved effective at FTAI during the non-breeding season [34,81]. A fertility rate of 48.9% was reported by [34], following cervical AI performed 24 h after the onset of oestrus, which was similar to the CIDR–eCG protocol under the conditions of this study. Accordingly, Ref. [81] worked with different flocks to develop hormone-free FTAI protocols based on ME. The workers characterised oestrus and ovulatory responses to ME over a 4-year period, with the aim of establishing the correct time for the application of AI. Artificial insemination performed at day 7 and 8 of buck exposure, regardless of heat expression, resulted in a kidding rate of 58% using frozen thawed semen. However, a higher kidding rate of 64% was achieved when goats were inseminated in a similar manner, after oestrus detection. In this study, the LH surge of the fertile ovulation, which should ideally be the target for the development of hormone-free AI protocols, occurred over a period of 48 h between 6 and 8 days after male exposure. The highest kidding rates (about 70%) were obtained when does were inseminated cervically within 24 h of the LH surge. Therefore, effective hormone-free protocols such as these, which can be associated with FTAI, are an excellent option for organic farm operations.

### 5.4. Use of Short-Term Nutritional Supplementation to Increase Ovulation Rate and Improve Embryo Quality and Fertility

**Figure 5:**
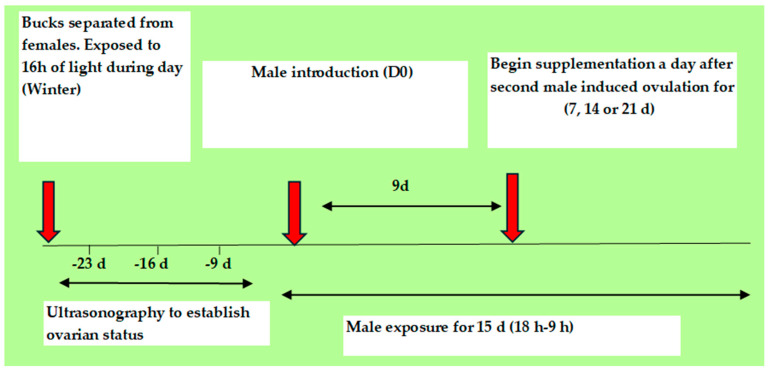
Schematic representation of an oestrous synchronisation protocol based the on use of photostimulated bucks combined with the short-term supplementation of females with a high energy–protein diet. (Based on the study of Fitz-Rodríguez et al. 2009 [85]).

Short-term nutritional supplementation can be accomplished using high energy diets [82] or infusions such as glycogenic mixtures (glycerol + propylene glycol + water) [41,83,84]. Other nutritional supplements include high protein-energy diets [85] and micronutrients such as betacarotene (β-carotene) [86,87] or fatty acids (FAs) [88,89]. When supplied at specific physiological stages, the metabolic status, follicle development, oocyte/embryo quality, or ovulation rate are influenced and, consequently, fertility is improved (Figure 5). Short-term energy supply causes an immediate increase in glucose [41,82,84,90] and metabolic hormones, such as insulin and leptin [82,84,91]. The presence of glucose transporter proteins and specific receptors of metabolic hormones in the ovarian tissues ensures the uptake and action of glucose directly at the ovarian level [91,92]. In ewes, this action is reported to cause a decrease in E2 concentration and negative feedback on the hypothalamus and anterior pituitary, which stimulates an increase in FSH and follicle development [93]. Extensive studies in sheep report an increase (*p <* 0.05) in the number of follicles of all classes (i.e., both small, 2–3 mm, and medium sized, 4 mm), growing to preovulatory-sized large follicles, at around the time of luteolysis/device withdrawal and after device withdrawal, in ewes supplemented with high energy compared with the non-supplemented groups [41,82,90]. An interesting observation, made by [90], was that a higher number of follicles growing from 2 to 3 mm (2.0 ± 0.4 vs. 0.9 ± 0.4, *p ≤* 0.05), and a higher (*p ≤* 0.05) number of ovulatory follicles of ≥6 mm, were detected in ewes supplemented with energy than those that were non-supplemented. This indicates that through its influence on follicle growth, nutritional supplementation has the potential to increase the proportion of ewes ovulating [90] and the number of double ovulations [82]. The effect of a prolonged lifespan of the last non-ovulatory follicle (7.8 ± 0.6 vs. 10.2 ± 0.6, *p ≤* 0.05), observed by [90], also signifies that, like eCG, high energy supplementation may increase the ovulation rate by preventing the occurrence of atresia.

The influence of high energy supplementation on the ovulation rate was reported by [41], who observed a higher ovulation rate (1.9 ± 0.1 vs. 1.3 ± 0.2, *p <* 0.05) in ewes supplemented with glycogenic mixtures than the non-supplemented group. The influence of supplementation of glycogenic mixtures on the ovulation rate in ewes reported in this study was possibly influenced through an increased ovulatory competence of preovulatory follicles, given the observation that the ovulation rate in the supplemented group was higher but the number of large follicles in both the supplemented and non-supplemented ewes detected at oestrus was similar. It is known that an increased ovulatory competence of preovulatory follicles results in an improved oocyte quality and fertility [14]. Despite the influence of glycogenic mixtures on ovarian follicular dynamics and the ovulation rate, the administration of high doses may affect homeostasis through their effect on blood cell (RBC) indices [84]. Use of a medium dose (2.9% and an NE_L_ of 0.6 Mcal/kg/d on a DM basis) resulted in a significant increase in the number of large follicles (2.8 ± 1.0 vs. 2.2 ± 0.4, *p <* 0.05) compared with eCG, without affecting RBC indices, but did not influence the ovulation rate. This observation suggests that like eCG, the use of glycogenic mixtures possibly affects the ovulation rate when administered in high doses. However, given the effect of administering high doses of glycogenic mixtures on RBC indices, there is little doubt that their utilisation to stimulate an increased ovulation rate would not pose a risk. Considering this limitation, the use of other forms of short-term energy supplies can be explored.

Apart from the influence of glycogenic mixtures on follicular dynamics and the ovulation rate [41,83,84], fertility traits such oocyte competence, fertilisation rate, and embryo quality can also be influenced through supplementation with high energy diets, [82,90], high protein–energy diets [83], fatty acids [88,89], or β-carotene [87]. It is important to note that body weight (BW) and BCS are not influenced by short-term energy [90] or micronutrient supplementation [86]. For this reason, interventions on the ovulation rate may not succeed if females are in a low body condition, given the association of a low body condition score (BCS) with a reduced magnitude of the surge of gonadotrophins [94]. On the other hand, a high BCS is associated with high levels of circulating FSH and a lower E2 during proestrus, which promotes the growth and selection of more gonadotrophin-responsive follicles due to a low negative feedback of E2 on FSH secretion [95]. This therefore suggests that females should be in a good body condition for short-term nutritional supplementation to stimulate an increase in the ovulation rate. Furthermore, for a significant effect of short-term energy supplementation on the ovulation rate to be achieved, nutrients have been supplied from the mid to late luteal phase, i.e., shortly before P4 withdrawal or luteolysis [82,90], with the timing designed in such a way that levels of glucose and metabolic hormones peak around the time of emergence of the ovulatory wave [90]. As for supplementation aimed at influencing embryo survival, nutrients are supplied beyond the luteal phase (i.e., through to the follicular phase to target the critical stage of pregnancy establishment [87]). For example, the pregnancy rate was influenced (*p* < 0.05) in anovulatory female goats under extensive grazing conditions, which exposed to ME for 15 days and fed a high protein diet for 7 days, starting 9 days after male introduction, compared with non-supplemented goats, although the effect was greater (*p* < 0.05) with a 14-day supplementation period (i.e., supplementation was provided for 7 more days after male exposure). In this study, a pregnancy rate above 80% was achieved in supplemented does compared with a pregnancy rate below 60% which was observed in the non-supplemented does [85].

There are reports that the supplementation of goats with micronutrients such as fatty acids or betacarotene combined with ES or superovulation protocols can influence the ovulation rate, conception rate, or embryo quality. In a study by [88], goats synchronised with double long-interval PGF2α injections were either supplemented with palm oil or n-3 polyunsaturated fatty acid (PUFA)-rich fish oil prior to mating. In addition to the increase in the obtained ovulation rate, which was 41.35% higher in the group supplemented with n-3 PUFA-rich fish oil than the control group, a tendency for an increased conception rate (85.71% vs. 76.92%, *p* = NS) was observed. In another study with FAs, inclusions of omega-3 and omega-6 fatty acids from linseed oil in the diet of Boer goats synchronised with a protocol to achieve superovulation resulted in a higher (*p* < 0.05) mean number of recovered ova/embryos and transferable embryos, in addition to the prevention of premature CL regression [89]. Embryo loss can also be prevented by β-carotene supplementation [87]. For example, β-carotene supplementation increased the P4 concentration and glutathione peroxidase activity in Saanen goats, which signifies that β-carotene could have a key role in improving fertility through the establishment of pregnancy and/or improved embryo survival. This is important, given that ES treatments are associated with high embryo loss during early pregnancy [94], which reduces fertility yields.

## 6. Conclusions and Recommendations

Based on the literature, the short-term CIDR protocol can be promoted for use in tropical and subtropical regions, particularly the 5-day CIDR treatment, which has demonstrated high fertility and can still produce adequate fertility yields when used thrice, compared with protocols which involve longer treatment durations. This affords a cheaper protocol, which is also more environmentally safe; therefore, it is a more sustainable protocol. Apart from the treatment duration and the re-use of CIDRs, the administration of eCG, PGF_2_α, and the time of PGF_2_α application also appear to induce a different pattern of follicular growth in females treated with the short-term CIDR protocol, which influences the timing of preovulatory events and ovulation, suggesting the need for readjustments in the timing of AI. Studies have also demonstrated that the use of high doses of eCG, particularly under tropical climatic conditions, is unnecessary. Therefore, with the observation that ME and short-term nutritional supplementation can influence fertility when combined with short-term CIDR, research should be undertaken to develop protocols for FTAI, which involve a combination of short-term CIDR, ME, and short-term nutritional supplementation, given the possibility that such protocols may influence ovulatory response and fertility the same way as eCG does, which in turn would eliminate the need to use this gonadotrophin.

## Data Availability

Not applicable.
